# A new scoring system and clinical algorithm for the management of suspected foreign body aspiration in children: a retrospective cohort study

**DOI:** 10.1186/s13052-021-01147-9

**Published:** 2021-09-28

**Authors:** Nader A. Fasseeh, Osama A. Elagamy, Alaa H. Gaafar, Heba M. Reyad, Mohamed S. Abougabal, Doaa A. Heiba, Ahmad Kantar

**Affiliations:** 1 Pediatric Respiratory and Allergy Unit, Faculty of Medicine, Alexandria University, Alexandria, Egypt; 2 Department of Pediatrics, Faculty of Medicine, Kafrelsheikh University, Kafrelsheikh, Egypt; 3 Department of Otolaryngology, Faculty of Medicine, Alexandria University, Alexandria, Egypt; 4 Pediatric Asthma and Cough Center, Istituti Ospedalieri Bergamaschi, Bergamo, Italy

**Keywords:** Foreign body aspiration, Bronchoscopy, Children, Clinical algorithm

## Abstract

**Background:**

Cases of foreign body aspiration in children may be encountered in emergency departments. A suggestive history is important in diagnosing aspirated foreign body owing to the difficulty in making a diagnosis on the basis of an abnormal physical examination or chest radiography alone. The aim of this study was to examine the sensitivity and specificity of the presenting symptoms, physical examination, and radiologic findings as predictors of foreign body aspiration in children. In addition, a feasible simple algorithm with a scoring system was generated to indicate bronchoscopic investigation.

**Methods:**

In a retrospective cohort, medical records of patients aged less than 16 years with suspected foreign body aspiration who underwent flexible or rigid bronchoscopy were included. Data including age, sex, symptoms, physical examination findings, radiological features, nature and location of the foreign body, and outcome of the bronchoscopy were collected, and multivariable binary logistic regression analysis was employed for prediction of foreign body aspiration.

**Results:**

A total of 203 children were included, and the model showed excellent discrimination power for positive foreign body aspiration (area under the curve = 0.911) with an accuracy, sensitivity, and specificity of 86.2, 90.6, and 76.6%, respectively. The total weighted risk score at a cut-off > 2 showed a significant good power of discrimination (area under the curve = 0.879), with a sensitivity of 79.9% and specificity of 84.4%. Accordingly, a clinical algorithm was recommended.

**Conclusions:**

The proposed scoring system and clinical algorithm might help in decision making with regard to the need and type of bronchoscopy in children presenting with potential foreign body aspiration. However, further prospective multicenter studies should be conducted to validate this scoring system.

## Background

Foreign body aspiration (FBA) is encountered in many instances in the pediatric emergency department with potential serious consequences [1]. In 2017, *the National Safety Council (Itasca-IL, USA)* categorized it as the main cause of accidental death during the first year of infancy and as the fifth cause of unintentional death among children aged 1–4 years [2].

FBA results in either complete or partial occlusion of the conducting airways, causing serious clinical events such as pneumonia, bronchiectasis, lung abscess, atelectasis, or even death [3]. The severity of these complications is presumably related to missed or delayed diagnosis and management [4].

Lack of a history of penetration syndrome corresponding to respiratory defense reflexes (expulsive cough and laryngeal spasm) in response to penetration by a foreign body (FB) may veil the physician’s suspicion. Moreover, radiologic abnormalities on chest radiograph may be nonspecific or even normal in 35% of cases [5].

Bronchoscopy, whether rigid or flexible, is the standard procedure to ascertain and manage FBA. The use of flexible or rigid bronchoscopy has been a matter of debate for a few decades without a global approach to determine the type that can be used in children with suspected FBA. The aim of this study was to establish, based on our experience, a feasible simple clinical algorithm with a scoring system to determine criteria for bronchoscopy in children with suspected FBA.

## Methods

### Study design and setting

A retrospective cohort study was conducted for 12 months at the El Shatby University Children Hospital and ENT department, Alexandria University (tertiary-level hospitals), starting from 1 April, 2018.

### Study population

All subjects aged less than 16 years who underwent rigid or flexible bronchoscopy for suspected FBA were enrolled.

### Study measures and data collection

The medical records of all recruited children were analyzed. Data obtained were categorized according to age, sex, conditions on admission, physical examination findings, radiological features and bronchoscopic findings, nature and location of the FB, and management outcomes.

Generally, any child attending to pediatric emergency with a suspected foreign body aspiration diagnosis is presented to a pediatric consultant. The child^’^s history, physical examination, and chest radiograph findings are evaluated. If there was a definite history of witnessed choking and the patient presented with significant physical examination findings such as hypoxia, tachypnea, reduced air entry, or obvious CXR abnormalities like radio-opaque FB, mediastinal shift, unilateral atelectasis or hyperinflation, or a combination of any of these, the patient was referred directly to the ENT consultant for urgent rigid bronchoscopy under general anesthesia. If the patient presented with more subtle radiological or physical examination findings, the pediatric pulmonology team was consulted to consider a flexible bronchoscopy as the initial procedure of choice.

### Anesthesia

Flexible bronchoscopy was performed under general anesthesia with spontaneous ventilation under continuous cardiorespiratory monitoring. The child was given a hypnotic dose of propofol (2 mg/kg) for induction of anesthesia with a maintenance infusion of 0.125–0.3 mg/kg/min to increase the depth of anesthesia. Anesthesia was maintained by the use of inhaled agents like sevoflurane. Atropine was administered at a dose of (0.01–0.02 mg/kg) in 20–30 min and midazolam (0.01–0.1 mg/kg) was injected 5–10 min before the procedure. Rigid bronchoscopy was performed under general anesthesia with administration of neuromuscular blocking agents such as succinylcholine to induce muscle relaxation. Flexible bronchoscopes, including video bronchoscope (Olympus 4.9 mm with working channel 2.2 mm and 4.2 mm with working channel 2 mm) and fibro scope (Karl Storz 2.8 mm and 3.7 mm with working channel 1.2 mm for both), and rigid bronchoscopes (Karl Storz 2.5 mm, 3 mm, 3.5 mm, 3.7 mm, 4 mm, 5 mm, and 6 mm) are employed.

### Statistical analysis

Statistical analysis and presentation of data were conducted using Statistical Package for the Social Sciences (SPSS) version 22. Categorical data are presented as numbers and percentages. The chi-square test was applied to investigate the association between categorical variables. Alternatively, the Fisher’s exact test was applied when the expected cell counts were less than 5. Continuous data were tested for normality using the Shapiro-Wilk test. It represented a non-normal distribution and was expressed as median and interquartile range (IQR) (25th–75th percentiles), and the Mann-Whitney U test was used for comparison. The diagnostic value of each variable significantly associated with positive FBA was investigated by receiver operating characteristic (ROC) curve analysis. A multivariable binary logistic regression analysis (backward stepwise method) was performed to determine the independent predictors of FBA from statistically significant variables with positive FBA as well as variables with a *p* value < 0.1. A weighted risk score for each predictor was calculated by dividing its beta coefficient by the smallest coefficient and then approximated to the nearest integer. The total weighted risk score for each patient was further calculated by summing all the predictor’s scores. The diagnostic performance of the score in predicting positive FBA was performed using ROC curve analysis. Finally, a clinical algorithm was suggested based on the frequency of a proven FBA among different categories of the total weighted risk score. A *p*-value of < 0.05 was considered statistically significant.

## Results

Medical records of 263 children who were admitted with a diagnosis of suspected FBA were investigated. Of these, 60 were excluded because of missing data. Of the 203 patients (age ranging between 4 months and 15 years, mean = 2.99 years), 134 were male (66.0%). FBA was most frequent in the age group 1–2 years, followed by 2–3 years (26.6 and 20.2%, respectively) with a median age of 2.6 years (IQR = 1.5–4.5). FBA was verified in 139 (68.5%) patients. In 126 (90.6%) cases, the FB was removed using rigid bronchoscopy, while 13 (9.4%) underwent diagnostic flexibility followed by rigid bronchoscopy for FB removal. Organic foreign bodies constituted 82.0% (*n* = 114) of all cases, whereas 18.0% (*n* = 25) were inorganic. Nuts were the most commonly retrieved organic FBs (*n* = 92, 80.7%), while scarf pin was the most frequently retrieved (*n* = 8, 32%) inorganic FB followed by a pen cap (*n* = 3, 12%). The right main bronchus was the most common (*n* = 59, 42.4%) location of the FB, followed by the left main bronchus (*n* = 29, 20.9%), and trachea (*n* = 26, 18.7%) (Table 1).

**Table 1:** Demographic and bronchoscopy findings

	N	%
Age groups (years)		
< 1	15	7.4%
1–2	54	26.6%
2–3	41	20.2%
3–4	27	13.3%
4–5	19	9.4%
5–6	10	4.9%
≥ 6	37	18.2%
Sex		
Female	69	34.0%
Male	134	66.0%
Foreign body aspiration		
No foreign body	64	31.5%
Foreign body present	139	68.5%
Type of foreign body		
Organic	114	82.0%
Inorganic	25	18.0%
Organic foreign body		
Nuts	92	80.7%
Seed	17	14.9%
Lupine	6	5.3%
Corn	5	4.4%
Fish bone	2	1.8%
Chicken bone	1	0.9%
Pasta	1	0.9%
Meat	1	0.9%
Inorganic foreign body		
Scarf pin	8	32.0%
Pen cap	3	12.0%
Paper	2	8.0%
Bead	2	8.0%
Metallic object	2	8.0%
Plastic piece	2	8.0%
Zipper	1	4.0%
Button	1	4.0%
Metallic Pen cap	1	4.0%
Plastic piece and metallic object	1	4.0%
Unidentified	2	8.0%
Bronchoscopy type		
Rigid	144	70.9%
Flexible	46	22.7%
Flexible then rigid	13	6.4%
Site of foreign body		
Right main bronchus	59	42.4%
Left main bronchus	29	20.9%
trachea	26	18.7%
Subglottic	17	12.2%
others	8	5.8%

Table 2 demonstrates significant associations between the presence of a FB in the airways and age, witnessed choking episodes, sudden cough onset, new onset or recurrent wheeze, and the presence of unilateral diminished breath sounds, respiratory distress, and wheezes on the chest examination. In addition, the manifestation of radiographic abnormalities, unilateral chest hyperinflation, or radiopaque shadows were significantly associated with positive FBA (*p* < 0.05).

**Table 2:** Association between demographic, clinical, and radiologic findings and the presence of foreign body

		Foreign body				
		Positive *N* = 139 (68.5%)		Negative *N* = 64 (31.5%)		
		N	%	N	%	*P* value
Age median (IQR)		2.0 (1.5–3.5)		3.8 (2.0–6.8)		0.001*
Sex	Female	47	33.8%	22	34.4%	0.937
	Male	92	66.2%	42	65.6%	
**Symptoms**						
Witnessed Chocking	No	64	46.0%	45	70.3%	0.001*
	Yes	75	54.0%	19	29.7%	
Noisy breathing/stridor/ hoarseness	No	126	90.6%	63	98.4%	0.082
	Yes	13	9.4%	1	1.6%	
Persistent or chronic cough	No	78	56.1%	31	48.4%	0.308
	Yes	61	43.9%	33	51.6%	
Sudden cough	No	118	84.9%	63	98.4%	0.004*
	Yes	21	15.1%	1	1.6%	
Hemoptysis	No	137	98.6%	63	98.4%	> 0.999
	Yes	2	1.4%	1	1.6%	
New onset\recurrent wheeze	No	103	74.1%	61	95.3%	< 0.001*
	Yes	36	25.9%	3	4.7%	
Recurrent lower respiratory tract infection	No	132	95.0%	57	89.1%	0.214
	Yes	7	5.0%	7	10.9%	
Dyspnea	No	116	84.1%	59	92.2%	0.114
	Yes	22	15.9%	5	7.8%	
Fever	No	134	96.4%	58	90.6%	0.175
	Yes	5	3.6%	6	9.4%	
Cyanosis	No	129	92.8%	63	98.4%	0.189
	Yes	10	7.2%	1	1.6%	
**Physical examination**						
Unilateral diminished breath sound	No	79	56.8%	60	93.8%	< 0.001*
	Yes	60	43.2%	4	6.3%	
Bilateral diminished breath sound	No	128	92.1%	61	95.3%	0.555
	Yes	11	7.9%	3	4.7%	
Wheezy chest	No	84	60.4%	49	76.6%	0.025*
	Yes	55	39.6%	15	23.4%	
Stridor	No	114	82.0%	59	92.2%	0.058
	Yes	25	18.0%	5	7.8%	
Respiratory distress	No	114	82.0%	61	95.3%	0.011*
	Yes	25	18.0%	3	4.7%	
Crepitation	No	133	95.7%	57	89.1%	0.138
	Yes	6	4.3%	7	10.9%	
Normal physical examination	No	106	84.1%	27	42.2%	< 0.001*
	Yes	20	15.9%	37	57.8%	
**Radiologic Findings**						
Abnormality	No	81	58.3%	48	75.0%	0.021*
	Yes	58	41.7%	16	25.0%	
Radiopaque FB	No	126	90.6%	64	100.0%	0.011*
	Yes	13	9.4%	0	0.0%	
Unilateral hyperinflation	No	124	89.2%	63	98.4%	0.023*
	Yes	15	10.8%	1	1.6%	
Bilateral hyperinflation	No	132	95.0%	62	96.9%	0.722
	Yes	7	5.0%	2	3.1%	
Consolidation/collapse	No	128	92.1%	59	92.2%	> 0.999
	Yes	11	7.9%	5	7.8%	
Infiltrates	No	129	92.8%	60	93.8%	> 0.999
	Yes	10	7.2%	4	6.3%	
Ground glass opacities and bronchiectasis	No	137	98.6%	60	93.8%	0.151
	Yes	2	1.4%	4	6.3%	

*significant at *p* < 0.05; IQR: interquartile range

The diagnostic performance of each of the aforementioned significant clinical and radiologic findings was investigated by ROC curve analysis, as shown in Table 3. All of them exhibited poor diagnostic power of FBA (areas under the curve [AUCs] ranged from 0.568 to 0.685).

**Table 3:** Diagnostic performance of the significant clinical and radiologic findings as predictors of foreign body aspiration

	Sensitivity %	Specificity %	+LR	-LR	AUC	95% CI of AUC	P value
Witnessed chocking	54.0	70.3	1.82	0.65	0.621	0.551 to 0.688	< 0.001*
Sudden cough	15.1	98.4	9.67	0.86	0.568	0.497 to 0.637	< 0.001*
New onset or recurrent wheezes	25.9	95.31	5.53	0.78	0.606	0.535 to 0.674	< 0.001*
Unilateral diminished breath sound	43.2	93.8	6.91	0.61	0.685	0.616 to 0.748	< 0.001*
Wheezy chest	39.6	76.6	1.69	0.79	0.581	0.510 to 0.649	0.017*
Respiratory distress	18.0	95.3	3.84	0.86	0.566	0.495 to 0.636	0.001*
Chest X-ray Abnormality	41.7	75.0	1.67	0.78	0.584	0.513 to 0.652	0.015*
Radiopaque FB	9.4	100.0	–	0.91	0.547	0.476 to 0.617	0.002*
Unilateral hyperinflation	10.8	98.4	6.91	0.91	0.546	0.475 to 0.616	0.002*

*significant at p < 0.05; AUC: area under the curve. +LR: positive likelihood ratio, −LR: negative likelihood ratio; CI: confidence interval

Multivariable binary logistic regression analysis was applied to retrieve a model for the prediction of FBA. Witnessed choking, sudden cough, new-onset or recurrent wheeze, unilateral diminished breath sounds, wheezy chest, respiratory distress, and radiographic findings of unilateral hyperinflation were the risk factors that significantly contributed to the model. The model significantly predicted FBA (× 2 = 109.91, *p* < 0.001), with an accuracy, sensitivity, and specificity of 86.2, 90.6, and 76.6%, respectively. The model was also fit as indicated by the Hosmer and Lemeshow model fit test of 0.0.097. The model showed excellent discrimination power for positive FBA (AUC = 0.911). The presence of unilateral diminished breath sounds was associated with a 33.73-fold increased likelihood of FBA. Finally, the weighted risk score for each predictor in this model was calculated according to its coefficient, as illustrated in Table 4.

**Table 4:** Multivariable binary logistic regression model and weighted risk score for prediction of foreign body aspiration

Variables	Coefficient	Weight risk score	AOR	95% CI of AOR	P value	R^2^%	Accuracy %	Sens.%	Spec.%	AUC	95% of AUC
Witnessed chocking	1.75	2	5.78	2.30–14.54	< 0.001*	58.7	86.2	90.6	76.6	0.911	0.863 to 0.946
New onset/recurrent wheeze	1.67	2	5.31	1.15–24.36	0.032*						
Sudden cough	3.08	3	21.71	2.30–204.63	0.007*						
Unilateral diminished breath sounds	3.52	3	33.729	9.36–121.53	< 0.001*						
Wheezy chest	1.10	1	3.012	1.19–7.63	0.020*						
Respiratory distress	2.28	2	9.774	2.07–46.09	0.004*						
Unilateral hyperinflation	2.59	2	13.338	1.09–162.42	0.042*						

*significant at p < 0.05; AOR: adjusted odds ratio, AUC: area under the curve; Sens.: sensitivity; Spec.: specificity; CI: confidence interval

**Fig. 1:**
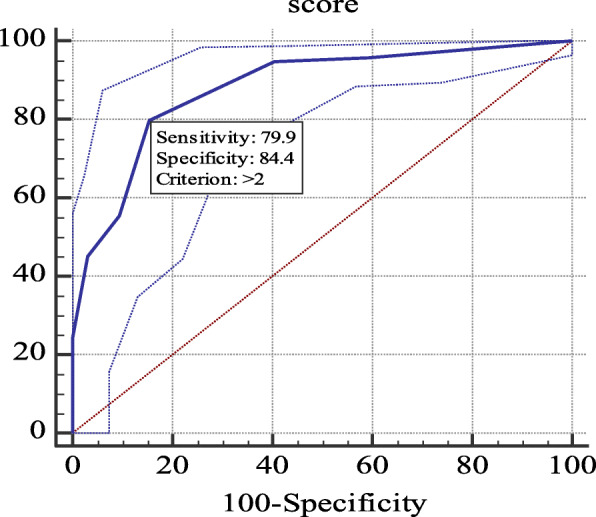
Receive operating characteristic for prediction of foreign body aspiration by the weighted risk score

The total weighted risk score which represents the sum of all the predictors scores ranged from 0.0 to 10.0, with a median of 3.0 (IQR = 2.0–5.0). At a cutoff > 2, the score showed significant good discrimination power of positive FBA (AUC = 0.879), with a sensitivity of 79.9% and a specificity of 84.4%, as shown in Table 5 and Fig. 1.

**Table 5:** Evaluation of the diagnostic value of the weighted risk score as predictor of foreign body aspiration

	Cut off	Sensitivity%	Specificity%	+LR	-LR	Accuracy%	AUC	95% CI of AUC	P value
Score	> 2	79.9	84.4	5.11	0.24	83.7%	0.879	0.826 to 0.921	< 0.001*

*significant at p < 0.05; AUC: area under the curve. +LR: positive likelihood ratio, −LR: negative likelihood ratio; CI: confidence interval

**Fig. 2:**
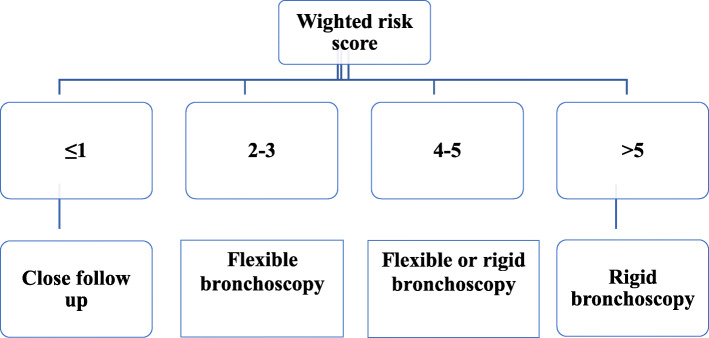
Clinical algorithm for bronchoscopy decision in children with suspected foreign body aspiration

Table 6 demonstrates a significant association between the weighted risk score and positive FBA (*p* < 0.001). FBA was proven in 15.6% of patients with a score ≤ 1, while positive FBA was observed in all (100%) patients with a score > 5. According to the suggested score, a clinical algorithm was recommended (Fig. 2) for bronchoscopy decisions in children with suspected FBA.

**Table 6:** The association between positive foreign body aspiration and the weighted risk score

			Weighted risk score					
			≤1	2–3	4–5	> 5	Total	*P* value
FBA	Positive	N	7	55	43	34	139	< 0.001*
		%	15.6%	73.3%	87.8%	100.0%	68.5%	
	Negative	N	38	20	6	0	64	
		%	84.4%	26.7%	12.2%	0.0%	31.5%	
	Total		45	75	49	34	203	

*significant at p < 0.05; FBA: foreign body aspiration

## Discussion

FBA may present a life-threatening emergency in children. It requires early diagnosis and urgent removal of FB by bronchoscopy to avoid complications [6]. In this study, we formulated a clinical algorithm based on an objective scoring system to standardize the management approach for children with suspected FBA. This simple algorithm may guide physicians in deciding whether to proceed with bronchoscopic intervention, ensuring prompt management of patients at a high risk for FBA and avoiding unwarranted investigations.

Our data indicate that thorough history taking, clinical examination, and conventional chest radiographic examination revealed a positive bronchoscopy rate of 68.5%. This percentage is much higher than that recently reported by Janahi et al. [7] (30.3%) and Özyüksel et al. [8] (47.1%). Earlier studies in children with suspected FBA showed a wide variation in the rate of positive bronchoscopy, from 25 to 90% [9–12]. This is most likely attributed to the lack of common standards that raise consistent decision making regarding bronchoscopy [7].

In this study, removal of FB was performed using rigid bronchoscopy in 90.6% of patients, while the remaining underwent diagnostic flexibility followed by rigid bronchoscopy. Moreover, rigid bronchoscopy is considered the gold standard for management of FBA. Nevertheless, this procedure has its own risks, including bronchospasm, edema of the airway, drop in oxygen saturation, and/or bleeding, as well as the additional risk of general anesthesia in children [13]. Hence, a realistic decision should be made to avoid unnecessary bronchoscopy procedures and their negative rates [8].

There has been a great effort to identify patients with FBA based on history, symptoms, physical findings, and radiologic investigations [8]. Thus, a significant association was observed between the history of the witnessed choking episode and verification of FBA. Despite the importance of this information as a clue for the diagnosis of FBA, it only exhibits an equivocal diagnostic sensitivity of 54.0%. Likewise, the absence of choking episodes does not rule out FBA. In contrast with our findings, a higher sensitivity (90.1%) of history for diagnosing FBA was reported by Kiyan et al. [14]. Alternatively, some recent studies reported the absence of a significant association between witnessed aspiration and FBA [8, 15].

The present study also revealed a significant association between reporting a novel onset or recurrent wheeze by the caregivers and positive FBA; however, it had a low sensitivity of 25.9%. A comparable finding of 22% was also reported by Janahi et al. [7]. Concerning physical examination findings, the presence of unilateral reduced breath sounds, respiratory distress, and wheezes on the chest examination also showed a significantly low diagnostic sensitivity for FBA with a value of 43.17, 17.99, and 39.57, respectively. Consistent with our findings, Özyüksel et al. [8] reported wheezing and decreased breath sounds at one site as the most frequent physical findings that significantly contribute to the prediction of FBA. Furthermore, choking and acute cough represented a sensitivity of 91.1% and specificity of 45.2% for diagnosing FBA [16]. However, a study by Kiyan reported a greater diagnostic role for physical findings, with much higher sensitivity (94.6%) [14].

Conventional plain chest radiograph is a diagnostic aid for FBA; however, its role is controversial [17]. In the current study, radiologic abnormalities including hyperinflation on one side (10.8%) and opaque foreign bodies (9.4%) were the most common and suggestive findings of FBA. Moreover, the diagnostic role of radiopaque FB was characterized by a low sensitivity of 9.4% and high specificity of 100.0%. Hence, it could be considered more helpful in excluding, rather than confirming, FBA. A retrospective review of a 10-year experience involving Mansoura University and Emergency Hospital reported radiopaque foreign bodies only in 23.56% of all patients with FBA [18]. Additionally, Silva et al. [19] reported a sensitivity and specificity of 73 and 45%, respectively for imaging studies in identifying FBA. A recent study suggested point of care ultrasound as an adjuvant to the standard assessment of suspected FBA in the emergency department. Neck examination using point of care ultrasound may detect foreign bodies outside the airways and it can reduce the time of bronchoscopy if needed [20], and it can explore indirect effects of lung inflammation as in aspiration pneumoniae [21].

To prevent serious complications of FBA, it is vital to diagnose and remove foreign materials promptly. The present work confirmed that discrete use of history, symptoms, signs, or radiologic findings was not sufficiently reliable for predicting FBA. For a more accurate diagnosis, multivariable regression analysis was performed to determine the most significant risk factors contributing to the positive presence of FB. It was found that a model of witnessed choking, sudden cough, new-onset or recurrent wheeze, unilateral diminished breath sounds, wheezy chest, respiratory distress, and X-ray findings of unilateral hyperinflation had an excellent diagnostic value with much higher sensitivity (90.6%) (Table 4). Similarly, Divarci et al. [22] demonstrated high sensitivity (91%) of combined use of positive history and clinical and radiologic findings in predicting FBA. Additionally, Sink et al. [23] reported that the chest wheezes and decreased breath sounds together increased the odds of FBA.

The development of a quantitative tool to assess children with potential FBA enables more accurate decision-making. Hence, one of the objectives of this study was to quantify the high-risk predictors of FBA, to develop a weighted risk score. The developed total weighted risk score had a minimum value of zero and a maximum of ten. Evaluation of its diagnostic performance revealed that a score higher than 2 was associated with a high likelihood of positive FBA with a sensitivity of 79.9% and specificity of 84.9% (Table 5). The application of this score is promising, as the rate of a missed FB and negative bronchoscopy would be minimized to about 20 and 15%, respectively. In comparison, Janahi et al. [7] proposed a score based on a comparable constellation of history, symptoms, signs, and radiologic findings. At a cutoff of ≥2, it showed a higher sensitivity (89.1%), but with a lower specificity (45.0%).

Our data demonstrate an increased percentage of proven FBA with increasing score. Inhalation of FB was proven in 15.6% of patients with a score ≤ 1, while positive FBA was observed in all (100%) patients with a score > 5 (Table 6). Accordingly, this feasible algorithm was proposed for deciding bronchoscopic intervention. In patients with a score > 5, the risk of FBA is very high, and rigid bronchoscopy is recommended. When a score of 4–5 was met, patients might be managed by either a flexible or rigid bronchoscopy depending on the hospital facilities and practitioner’s expertise. A lower score of 2–3 should be managed by a flexible bronchoscopy, as the expected rate of negative FBA would be high. Hence, these patients avoided the risks of rigid bronchoscopy. Finally, patients who scored < 1 can be safely discharged on the basis of availability of outpatient follow-up and close monitoring. Limited algorithms have been previously proposed to aid in the accurate diagnosis of FBA in children [7, 24]. An earlier study proposed a computerized scoring system consisting of 21 parameters. It showed a sensitivity and specificity of 95 and 70%, respectively [25]. A recent validation of this score system on 100 children with suspected FBA showed sensitivity of 100% and specificity of 41% [26]. Scoring systems and clinical algorithms might contribute to decision-making for bronchoscopy in children presenting with suspected FBA. Further studies to confirm our findings are needed from other independent centers with recruitment of much larger number of patients. Prospective studies to validate our score algorithm were scheduled in our hospital.

### Limitations

The current study had some limitations because it was a single-center experience with limited number of patients included as well as its retrospective nature, which might affect the availability and completeness of the data.

## Conclusions

The proposed scoring system and clinical algorithm might help in decision making with regard to the need and type of bronchoscopy in children presenting with potential foreign body aspiration. However, further prospective multicenter studies should be conducted to validate this scoring system.

## Data Availability

The datasets used and/or analyzed during the current study are available from the corresponding author on reasonable request.

## Abbreviations

AUC: Area under the curve
FBA: Foreign body aspiration
IQR: Interquartile range
ROC: Receiver operating characteristic
